# Surface chelation of cesium halide perovskite by dithiocarbamate for efficient and stable solar cells

**DOI:** 10.1038/s41467-020-18015-5

**Published:** 2020-08-25

**Authors:** Jingjing He, Junxian Liu, Yu Hou, Yun Wang, Shuang Yang, Hua Gui Yang

**Affiliations:** 1 Key Laboratory for Ultrafine Materials of Ministry of Education, Shanghai Engineering Research Center of Hierarchical Nanomaterials, School of Materials Science and Engineering, East China University of Science and Technology, 130 Meilong Road, 200237 Shanghai, China; 2 Centre for Clean Environment and Energy, School of Environment and Science, Gold Coast Campus, Griffith University, Brisbane, QLD 4222 Australia

**Keywords:** Energy, Solar energy, Solar cells

## Abstract

Surface engineering has been shown critical for the success of perovskite solar cells by passivating the surface enriched defects and mobile species. The discovery of surface modulators with superior interaction strength to perovskite is of paramount importance since they can retain reliable passivation under various environments. Here, we report a chelation strategy for surface engineering of CsPbI_2_Br perovskite, in which dithiocarbamate molecules can be coordinate to surface Pb sites via strong bidentate chelating bonding. Such chelated CsPbI_2_Br perovskite can realize excellent passivation of surface under-coordinated defects, reaching a champion power conversion efficiency of 17.03% and an open-circuit voltage of 1.37 V of CsPbI_2_Br solar cells. More importantly, our chelation strategy enabled excellent device stability by maintaining 98% of their initial efficiency for over 1400 h in ambient condition. Our findings provide scientific insights on the surface engineering of perovskite that can facilitate the further development and application of perovskite optoelectronics.

## Abstract (web summary)

Surface engineering is a known strategy to optimize the perovskite solar cells but it is usually based on weak bondings, such as Van der Waals, or coorordiating interacterions. Here He et al. report a chelation strategy using strongly adsorbed dithiocarbamate molecules and achieve high efficiency of 17.03% with excellent stability for CsPbI_2_Br based solar cells.

## Introduction

Lead halide perovskite-based solar cells are one of the most promising photovoltaic technologies benefited from its high efficiency, low cost, and solution processibility^1–6^. Unfortunately, the hybrid perovskites are susceptible to be degraded under thermal condition due to the volatility nature of the organic cations^7–9^. Replacing the volatile organic cations with inorganic Cs^+^ can offer intrinsically thermal stable perovskite phase over 400 °C with a tunable bandgap between 1.73 eV of CsPbI_3_ to 2.25 eV of CsPbBr_3_^10,11^. Among various cesium halide perovskites, CsPbI_2_Br perovskite is considered to be a good candidate for the high efficiency and stable all-inorganic perovskite solar cells (PSCs) due to its reasonable Goldschmidt tolerance factor and the lower phase transition temperature with band gaps between 1.82 and 1.92 eV^12,13^. Currently, the power conversion efficiency (PCE) of over 16% has been achieved for the CsPbI_2_Br solar cells, but their moisture and light instability is still an outstanding issue that remained to be solved^14,15^.

The degradation of perovskites has been identified to be initialized from the surfaces and/or grain boundaries, where are enriched with undercoordinated ions or mobile species^16–18^. These defective surfaces also introduce electronic trap states that act as fast channels for the nonradiative charge recombination^19^. Therefore, numerous studies have highlighted the significance of the passivation of surface defects and grain boundaries for achieving efficient and stable PSCs^20–25^. For example, fullerene and its derivatives can accept one electron from Lewis base type defects to passivate the perovskite surfaces in early studies^26,27^. Lewis bases, such as *π*-conjugated 6TIC-4F and theophylline molecules, have been employed to not only passivate the uncoordinated surface of the perovskite, but also better align the interfacial energy levels^15,28^. Most recently, oxysalts have been reported as surface inorganic passivation layers that can suppress ion migration and enhance the device stability by the merit of strong primary ionic bonding between lead cations of perovskite and sulfate anions^29^. In principle, the reliable and stable surface passivation for perovskites can be achieved through the formation of the strong chemical bond after passivation since its dissociation becomes difficult under various environmental stimuli. Among various bonding types, the chelation, that is embodied by a special bonding mode of polydentate molecules to central metal ions, can enable enhanced affinity to the perovskite surface than that of conventional nonchelating (monodentate) ligands^30,31^.

In this paper, we introduce the diethyldithiocarbamate (DDTC) molecule as a chelating agent for the surface engineering of CsPbI_2_Br perovskite. It was found that DDTC molecule strongly coordinates to surface Pb cation of perovskite via a bidentate chelating bonding. Such chelating structure enabled excellent and persistent passivation of surface defects of CsPbI_2_Br perovskite, generating significantly enhanced efficiency of 17.02% for CsPbI_2_Br solar cells as well as increased humidity and irradiation stability.

## Results

### Surface chelation of cesium halide perovskite

**Fig. 1:**
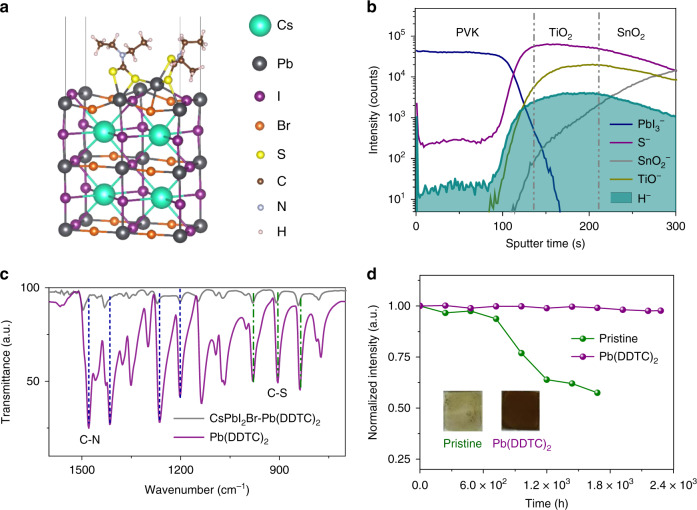
Chelation characterization and moisture tolerance study. **a** Atomic structure of optimized CsPbI_2_Br (001) surface with Pb(DDTC)_2_ molecule. **b** ToF-SIMS depth profile of the CsPbI_2_Br perovskite (PVK) film with Pb(DDTC)_2_ (chelated CsPbI_2_Br), measured in negative polarity. The measured film was structured as glass/FTO/c-TiO_2_/perovskite. The concentration of Pb(DDTC)_2_ used for the chelated sample is 0.015 M. **c** FTIR spectra of Pb(DDTC)_2_ (purple) and chelated CsPbI_2_Br (gray) samples. The concentration of Pb(DDTC)_2_ and CsPbI_2_Br perovskite in precursor solution are 0.02 and 1.0 M, respectively. C–N stretching and C–S stretching were highlighted by blue and green colored dotted line, respectively. **d** Evolution of normalized absorbance for pristine (green) and chelated (purple, Pb(DDTC)_2_) CsPbI_2_Br thin films under dark in ambient atmosphere (RH, 15 ± 3%). Absorption intensities at the wavelength of 400 nm were extracted from the UV–Vis absorption spectra of CsPbI_2_Br films. Insets are the photographs of pristine and chelated CsPbI_2_Br films after exposure to ambient air for 70 and 95 days, respectively.

Figure 1a shows the optimized CsPbI_2_Br (001) surface associated to lead diethyldithiocarbamate (Pb(DDTC)_2_) molecule, in which two –NCS_2_ groups anchor at central Pb ion with a bidentate configuration (see the atomic structure in Supplementary Fig. 1)^30,31^. The theoretical results based on the density functional theory (DFT) reveal that the Pb(DDTC)_2_ is adsorbed strongly on the surface with an adsorption energy of −1.73 eV, which is much larger than that of the prevalent passivation molecules, such as ammoniums or carboxylic acids of about −0.4 and −0.3 eV^32,33^. As a further comparison, the adsorption energy of H_2_O on the CsPbI_2_Br (001) surface is calculated to be only about one-fifth of it (−0.32 eV), which indicates the much weaker adsorption strength of water to perovskite (Supplementary Fig. 2a). In addition, positively charged Pb atom from Pb(DDTC)_2_ can be bonded strongly with surface Br and I atoms to neutralize the surface charge. However, the adsorption energy of PbI_2_ on CsPbI_2_Br (001) surface was only −1.11 eV (Supplementary Fig. 2b), which suggests that the chelation between DDTC and surface undercoordinated Pb can further strengthen the adsorption. When DDTC anions bond to metal centers, they typically have serval coordination modes, such as isobidentate, anisobidentate, monodentate, triconnective (Supplementary Fig. 3)^31,34^. We notice that the DDTC molecules retain a bidentate chelating bonding to the center Pb cation in Pb(DDTC)_2_, yet offers extra interaction to another surface Pb atom, which causes the superior adsorption strength. Since the undercoordinated atoms in the pristine perovskite surface are typically the active sites for water adsorption and act as the electronic trap sites, our results suggest that the chelation of CsPbI_2_Br perovskite surfaces by using Pb(DDTC)_2_ can be a promising approach to stabilize them under humid environment and enhance the overall performance.

According to the theoretical prediction, we employed Pb(DDTC)_2_ as an additive in the perovskite precursor solution. The chelating agent is well dissolved in perovskite precursor solution, albeit insoluble in water (Supplementary Fig. 4). The addition of Pb(DDTC)_2_ with the concentration below 0.015 M almost do not change the surface morphology and roughness of the CsPbI_2_Br films as confirmed by the scanning electron microscopy (SEM) and the AFM images (Supplementary Figs. 5 and 6). X-ray diffraction (XRD) patterns in Supplementary Fig. 7 reveal the α-phase perovskite structure of all samples without notable shift of diffraction peaks. This implies that the added DDTC molecules might be distributed at the grain boundaries of perovskite because of its large size^26^. Time-of-flight secondary-ion mass spectrometry (ToF-SIMS) further shows strong H^−^ and S^−^ signals at the perovskite surface and the perovskite/TiO_2_ interface, while their intensities are less than a tenth of that at the interface throughout the perovskite film (Fig. 1b). The ToF-SIMS result presented here thus corroborates the enrichment of DDTC on the surface or interface of the polycrystalline perovskite film. In addition, the XPS results confirm the absence of Sin bare TiO_2_/FTO substrate (Supplementary Fig. 8). Therefore, the S^−^ signal in the TiO_2_ and FTO region of ToF-SIMS spectra should be stemmed from DDTC molecules.

Fourier transform infrared (FTIR) spectra of CsPbI_2_Br perovskite with Pb(DDTC)_2_ (chelated CsPbI_2_Br perovskite) show similar characteristic absorption peaks with that of Pb(DDTC)_2_, which ascertains the existence of chelating agents in perovskite after thermal annealing at 160 °C (Supplementary Fig. 9). The FTIR absorption bands at 830–1000 cm^−1^, 1450–1580 cm^−1^, and 2850–3000 cm^−1^ regions primarily correspond to stretching vibration of C–S, C–N, and C–H of DDTC molecules, respectively (Fig. 1c)^34,35^. The C–N bond of DDTC molecules for both samples belongs to thioureide from with partial double bond character, as the *v*(C–N) locates between *v*(C=N) (1640–1690 cm^−1^) and *v*(C–N) bands (1250–1350 cm^−1^) (Supplementary Fig. 10)^34,36^. The occurrence of *v*_as_ band (1067 cm^−1^) and *v*_s_ band (980 cm^−1^) of –CS_2_ of chelated perovskites reveals the bidentate nature of the dithiocarbamate moiety with slight geometrical distortion^35,36^. Compared with Pb(DDTC)_2_, the *v*(C–N) and *v*(C–S) of the chelated CsPbI_2_Br perovskite shifted from 1481 and 904 cm^−1^ to 1497 and 910 cm^−1^, respectively, suggestive of the enhanced electron density near C–N bond of DDTC molecule, which is also supported by the change of Pb–S vibration peak (300–400 cm^−1^) from Raman spectra (Supplementary Fig. 11). Furthermore, ^13^C-NMR spectra in Supplementary Fig. 12 show that carbon signal of –NCS_2_ group of DDTC with CsPbI_2_Br shifted to high-field by Δ*δ* = 2.0 ppm compared to that of pure Pb(DDTC)_2_, in consistent with FTIR and Raman results. This behavior might be stemmed from the electron donation from the extra unsaturated Pb sites of perovskite surface as suggested by the DFT simulation^37–39^. Orthogonal FTIR measurements further reveal the dependence of C–N stretching of DDTC molecules on the targeted materials: *v*(C–N) of DDTC molecules with CsI, PbI_2_ and CsPbI_2_Br are at 1481, 1495, and 1495 cm^−1^, respectively (Supplementary Fig. 13). The shift of *v*(C–N) implies that the DDTC molecules mainly coordinate to Pb ions from PbI_2_ or perovskite, other than Cs ions from CsI that can be explained by Pearson acid–base concept, in good consistence with ^13^C-NMR results.

The surface chemical states of halides of perovskite were characterized by X-ray photoelectron spectroscopy (XPS). The I 3*d* spectra exhibit two contributions, 3*d*_5/2_ and 3*d*_3/2_, located at 618.5 and 629.8 eV for pristine film. The Br 3*d*_5/2_ spectra exhibit two peaks at 68.4 and 69.4 eV, respectively (Supplementary Fig. 14)^40^. Inclusion of Pb(DDTC)_2_ decreases the binding energies of I 3*d*_5/2_ and Br 3*d*_5/2_ to be 618.2 and 68.2 eV, respectively, which infers enhanced electron density around surface Br/I species. This can be explained as the reduction of undercoordinated halide ions on perovskite surface through the interaction with the Pb cations in Pb(DDTC)_2_^29,40^. The downshifted Pb 4*f* peaks, as well as the emergence of and S 2*s* peak of the chelated film, indicating the formation of Pb-DDTC bonding at the surface^15,28^. Based on above structural and electronic properties combined with the theoretical results, we conclude that the DDTC molecules chelate surface Pb cations via a bidentate configuration to passivate surface unsaturated Pb sites. Moreover, the chemical bonding between surface chelated Pb cations and surface I/Br anions should be reinforced, which may further reduce the surface undercoordinated halide related defects^37^.

The CsPbI_2_Br thin films with and without Pb(DDTC)_2_ were then stored in the ambient environment with relative humidity (RH) of 15 ± 3% for 95 days. The chelated CsPbI_2_Br film kept the original color over the entire tests, whereas the pristine one gradually turned yellow at 960 h (Supplementary Fig. 15). The UV–Vis spectra present the stable light absorbance of the chelated film in ambient air over 95 days (Fig. 1d and Supplementary Fig. 14b). Furthermore, the water contact angles of CsPbI_2_Br films increase from 42 to 76° after chelation, which indicates the reduction of surface hydrophilicity by surface chelation (Supplementary Fig. 16).

### Photovoltaic performance

**Fig. 2:**
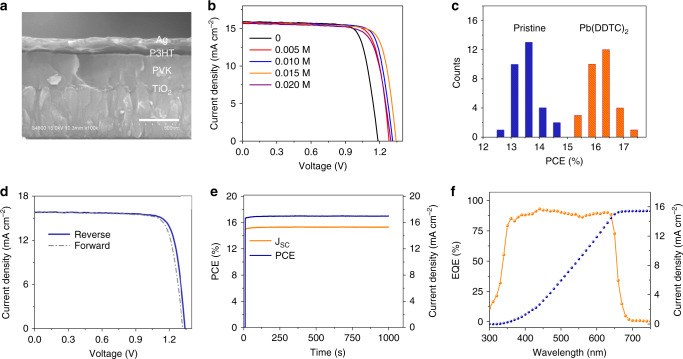
CsPbI_2_Br PSCs characterizations. **a** Cross-sectional SEM image of a chelated perovskite device. Scale bar is 300 nm. **b** *J*–*V* curves of perovskite solar cells with different concentrations of Pb(DDTC)_2_ under simulated AM 1.5G illumination (100 mW cm^−2^). **c** PCE histograms of pristine (blue) and chelated (orange) CsPbI_2_Br PSCs. **d** *J*–*V* characteristics of the champion device for chelated CsPbI_2_Br solar cell at opposite scan directions. **e** Steady-state power output (blue) and current density (orange) of the champion cell measured at a fixed maximum power point (MPP) voltage (1.11 V) as a function of time. **f** EQE spectra (orange) and corresponding integrated photocurrent (blue) of the champion device.

The cross-sectional SEM image of the typical planar heterojunction solar cell device architecture of glass/fluorine-doped tin oxide (FTO)/compact TiO_2_ (c-TiO_2_)/CsPbI_2_Br/P3HT/Ag is shown in Fig. 2a. The current density–voltage (*J*–*V*) curves of CsPbI_2_Br devices with different concentration of Pb(DDTC)_2_ measured under simulated AM 1.5G illumination are shown in the Fig. 2b. The corresponding photovoltaic parameters are summarized in Supplementary Table 1. The pristine device delivered a short-circuit current density (*J*_SC_) of 15.88 mA cm^−2^, an open-circuit voltage (*V*_OC_) of 1.19 V, a fill factor (FF) of 77.28% and a PCE of 14.54%. The addition of Pb(DDTC)_2_ mainly enhanced the *V*_OC_ of devices. The *V*_OC_ of the CsPbI_2_Br devices with 0.005, 0.010, 0.015, and 0.020 M Pb(DDTC)_2_ molecules were 1.28, 1.31, 1.34, and 1.30 V, generating high PCEs of 15.82%, 16.34%, 16.57%, and 15.31%, respectively. The reduction of PCE with excess Pb(DDTC)_2_ molecules can be explained by the large series resistance of insulating molecules. Given that *V*_OC_ are determined by quasi-Fermi level splitting under light irradiation, the chelated device should adopt much higher carrier concentration at open circuit probably because of the defect passivation effect of DDTC molecules. By using 0.015 M Pb(DDTC)_2_, we improved the average PCE from 13.70 ± 0.45% for pristine devices to 16.10 ± 0.44% for chelated ones (Fig. 2c). The champion chelated device showed a *V*_OC_ of 1.34 V, a *J*_SC_ of 15.78 mA cm^−2^, and a FF of 80.52%, generating a high PCE of 17.03% without notable hysteresis (Fig. 2d and Supplementary Table 2). This champion device is among the best reported CsPbI_2_Br ones (Supplementary Table 3). Moreover, a record *V*_OC_ of 1.37 V for chelated CsPbI_2_Br cell was also attained with the *V*_OC_ deficit of about 0.51 eV (Supplementary Fig. 17). The stabilized power output of champion device was recorded as 17.01% at maximum power point (MPP) under a bias of 1.11 V, in conjunction with the stable *J*_SC_ of 15.32 mA cm^−2^ after 1000 s (Fig. 2e). The integrated current density *J*_SC_ value obtained from the external quantum efficiency (EQE) measurements carried out on this device was calculated to be 15.43 mA cm^−2^, well matches to that obtained from *J*–*V* curve (Fig. 2f).

**Fig. 3:**
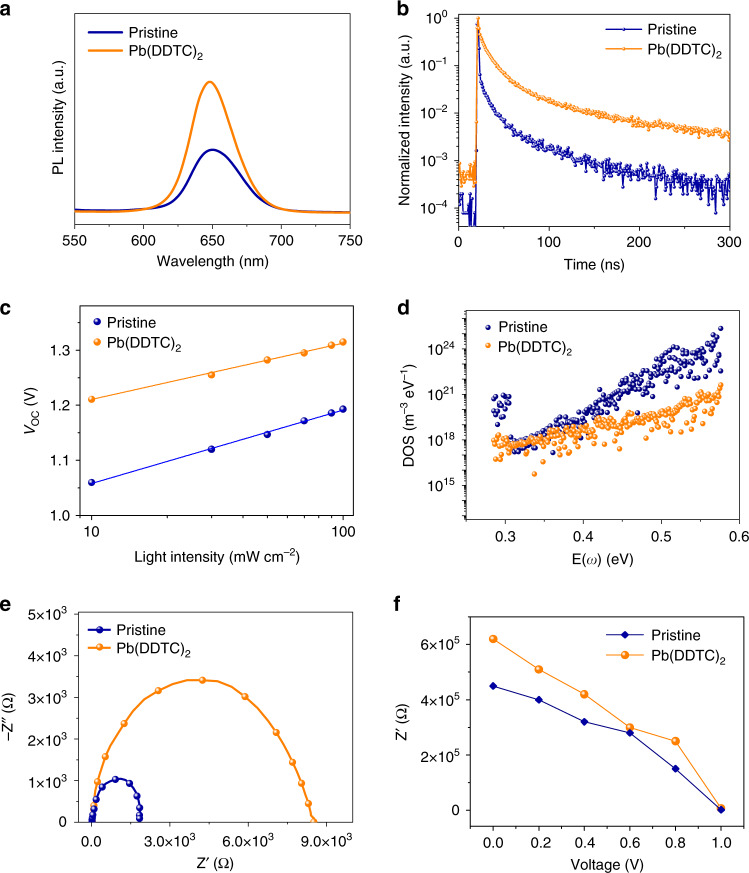
Performance characterizations of CsPbI_2_Br films and solar cells. **a**, **b** Steady-state PL and TRPL spectra, respectively, of the pristine (blue) and chelated (orange) CsPbI_2_Br film deposited on glass substrate. **c** Plots of light intensity dependent *V*_OC_ of the pristine (blue) and chelated (orange) CsPbI_2_Br devices. **d** tDOS of the pristine (blue) and chelated (orange) CsPbI_2_Br devices extracted from thermal admittance spectroscopy. **e** Nyquist plots of the pristine (blue) and chelated (orange) CsPbI_2_Br devices under dark with a bias voltage of 1.0 V. **f** Dependence of *R*_rec_ on applied bias voltage for pristine (blue) and chelated (orange) CsPbI_2_Br device. *R*_rec_ was obtained by fitting the EIS spectra at different voltages.

Steady-state photoluminescence (PL) and time-resolved PL (TRPL) measurements were operated, which are typical methods to assess the electronic passivation effect of chelating molecules on CsPbI_2_Br films^4,29,41^. As shown in Fig. 3a, the PL intensity of chelated film is enhanced to ∼2.1 times larger than that of pristine one, indicating the suppression of nonradiative charge recombination^25^. Meanwhile, the slight blue-shift of PL peak for the chelated film confirms the reduced shallow defects. TRPL spectra of both samples manifested a biexponential decay composed of a fast and a slow component that are typically assigned to charge trapping process and carrier recombination process, respectively (Fig. 3b)^23^. The fast decay lifetime (*τ*_1_) and slow decay lifetime (*τ*_2_) are 11.1 and 68.2 ns for the pristine CsPbI_2_Br film, and 13.3 and 91.3 ns for the chelated one, respectively. The high PL intensity and prolonged lifetime of the chelated film are associated with the decrease of electronic trap density^42^. The lower dark current density of chelated device further indicates the reduction of shunt pathways, which may originate from the defective grain boundaries (Supplementary Fig. 18). To further evaluate the recombination behavior of the solar cell devices, the dependence of *V*_OC_ on light intensities was measured and the results were plotted as a function of light intensity in logarithm scales as shown in Fig. 3c. Ideality factor (*n*) of the devices can be deduced by the slope of *V*_OC_ as a function of light intensity (*I*) according to the following equation^15^:

1 $$\begin{array}{*{20}{c}} {V_{{\mathrm{OC}}} = \frac{{E_g}}{q} - \left( {\frac{{{nkT}}}{q}} \right){\mathrm{ln}}\left( {\frac{{I_0}}{I}} \right)} \end{array},$$

where *E*_g_ is the bandgap of light absorber, *k* is the Boltzmann constant, *T* is the absolute temperature, *q* is the electron charge, and *I*_0_ is a constant with the same unit as *I*. Figure 3c shows the slopes of 1.76 *kT*/*q* for the chelated device and 2.22 *kT*/*q* for the pristine device, indicating the suppressed trap-induced recombination of the chelated device. Thermal admittance spectroscopy, as a well-established measurement of device trap density, was operated to measure the trap density of states (tDOS) of the as-fabricated devices^29^. As evidenced by Fig. 3d, the tDOS of chelated device are more than ten times lower than the pristine one in the deep trap region (greater than 0.50 eV). We speculate that the DDTC molecule passivate the surface undercoordinated Pb and halide ions as discussed above. Electrochemical impedance spectroscopy (EIS) characterization was performed to further probe the carrier dynamics of CsPbI_2_Br devices. The equivalent circuit model and fitting parameters of the impedance spectroscopy are showed in the Supplementary Fig. 19^43,44^. The recombination resistance (*R*_rec_) for the chelated device is about 4.3 times of that for pristine device at bias voltage of 1.0 V (Fig. 3e). The higher *R*_rec_ of the chelated device under different bias voltages further represents the longer carrier lifetime of the chelated device, benefiting from the reduction of surface trap states (Fig. 3f)^21,44^.

### Long-term device stability

**Fig. 4:**
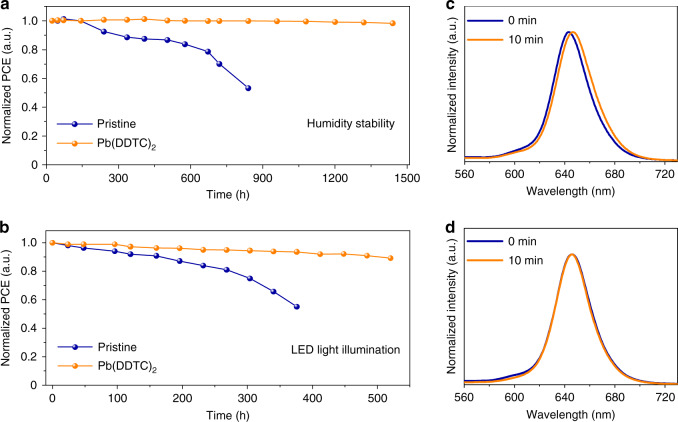
Stability performance of PSCs. **a** Evolution of power conversion efficiency of the pristine (blue) and chelated (orange) CsPbI_2_Br devices without encapsulation. The devices were stored under ambient atmosphere with controlled humidity (RH, 15 ± 3%). **b** Long-term stability of the pristine (blue) and chelated (orange) devices under continuous 100 mW cm^−2^ white LED illumination in nitrogen glovebox. All devices configurations are FTO/c-TiO_2_/perovskite/P3HT/Au. Normalized PL spectra of the (**c**) pristine and (**d**) chelated CsPbI_2_Br films under continuous one sun equivalent illumination for 10 min. All perovskite films were deposited on glass substrates.

Furthermore, the long-term stabilities of solar cell devices were tested under different conditions by adopting the device configuration of FTO/c-TiO_2_/perovskite/P3HT/Au. As given in Fig. 4a, the humid stability of chelated device was noticeably enhanced, maintaining over 98% of its original PCE under ambient conditions with 15 ± 3% humidity for 1440 h. In striking contrast, the pristine device only retained 53% of its initial efficiency within 840 h under the same conditions. The operational stability of CsPbI_2_Br devices presented in Fig. 4b indicated that there was only 11% efficiency drop for the chelated device after 520 h of continuous one-sun illumination in nitrogen atmosphere. As comparison, the pristine device lost 45% of its initial efficiency within 370 h. Moreover, the chelated device showed a slow decrease of PCE over time and maintained 90% of its initial efficiency after 450 h at 85 °C, while the PCE value of the pristine CsPbI_2_Br device dropped dramatically to 57% for 360 h (Supplementary Fig. 20). Above results have verified the essential role of Pb(DDTC)_2_ that stabilize the perovskite device from the degradation. Ions migration has been proved to be as a possible origin of devices’ intrinsic degradation under the working condition, which can be detected by PL emission under continuous light irradiation^15,26^. The PL emission peak of pristine CsPbI_2_Br thin film had a red shift of about 3 nm under the continuous light illumination for 10 min (Fig. 4c). Encouragingly, we found that the chelated film displayed no change in PL emission, suggesting the suppressed ion migration after chelation (Fig. 4d).

## Discussion

In summary, we have demonstrated the effectiveness of chelating molecular modulation on the surface property of perovskite films. Our experimental and theoretical results have elucidated the detailed surface chelation interactions of DDTC molecules to the surface Pb cations, which can passivate both undercoordinated surface Pb and I/Br defects. The champion PCE and *V*_OC_ of chelated CsPbI_2_Br cells attained 17.03% and 1.37 V, respectively, which are among the best reported results. Considering the strong adsorption of chelating molecules on perovskite, the stable bonding of chelating agents to the surface enables the long-term stability of perovskite devices. Other chelating agents, such as oxalic acid and ethylenediaminetetraacetic acid, were also tested with positive results. Our findings offer scientific avenues toward efficient and stable perovskite-based photovoltaic as well as other optoelectronic applications.

## Methods

### Materials and solution preparation

Lead nitrate (Pb(NO_3_)_2_, 99%), ethanol (≥99.7%) and sodium N,N-diethyldithiocarbamate trihydrate (NaDDTC·3H_2_O, 99%) were purchased from Sinopharm Chemical Reagent Company. Lead bromide (PbBr_2_, 99.9%), lead iodide (PbI_2_, 99.9%), cesium iodide (CsI, 99.9%), dimethyl sulfoxide (DMSO, anhydrous, ≥99.8%), dimethyl sulfoxide-*d*_6_ (DMSO-*d*_6_, ≥99.9%), chlorobenzene (≥99.9%), acetonitrile (≥99.8%), 4-tert-butylpyridine (t-BP, ≥96.0%), bis(trifluoromethane)sulfonimide lithium salt (Li-TFSI, ≥98.0%) and titanium(IV) chloride (TiCl_4_, >98%) were purchased from Sigma–Aldrich. Poly (3-methylthiophene) (P3HT) was purchased from Xi’an Polymer Light Technology Corp. All the chemicals and solvents were used as received without further purification. The Pb(DDTC)_2_ was synthesized by adding 20.0 M NaDDTC·3H_2_O aqueous solution of Pb(NO_3_)_2_ aqueous solution (10.0 M) dropwise under continuous magnetic stirring at room temperature. After stirring for 30 min, the yellow precipitate was filtered and washed four times with pure water and dried in an oven at 60 °C. Firstly, 30.22 mg Pb(DDTC)_2_ was dissolved into 2.0 mL DMSO solution to form Pb(DDTC)_2_-DMSO solution (0.03 M). Then, 235.5 mg PbI_2_, 187 mg PbBr_2_, and 272 mg CsI were dissolved in DMSO and Pb(DDTC)_2_-DMSO mixed solution to a final volume of 1 mL with desired Pb(DDTC)_2_ concentration, followed by stirring at room temperature overnight without filtration in a N_2_ glovebox.

### Device fabrication and characterization

FTO substrate (8 Ω per square, Nippon Sheet Glass) was sequentially cleaned with detergent, deionized water, acetone, and alcohol under sonication. Dry substrate was then immersed in a 25 mM TiCl_4_ aqueous solution for 60 min at 70 °C and washed with deionized water and ethanol, followed by annealing at 500 °C for 60 min in muffle to form a c-TiO_2_ blocking layer. The CsPbI_2_Br layer was fabricated by spin-coating the precursor solution on the FTO/c-TiO_2_ substrate via a two-step process with 500 rpm for 3 s, and 3500 rpm for 30 s. Subsequently, these films were annealed at 43 °C for 2 min, and then at 160 °C for 10 min^45^. P3HT (15 mg/mL in chlorobenzene, with 45 µL Li-TFSI solution (2 mg in 1 mL acetonitrile) and 10.2 µL t-BP) was spin-coated onto the CsPbI_2_Br perovskite film at 2500 rpm for 25 s as a hole transporting layer. Finally, the device was finished by evaporating Ag or Au layers^29^.

FTIR spectroscopy, Raman spectroscopy (Raman) and Carbon-13 nuclear magnetic resonance (^13^C-NMR) were performed by FTIR Nicolet 6700, Laser Raman InVia Reflex and Bruker Avance 500, respectively. The surface morphology and roughness of films were characterized by field emission scanning electron microscopy (HITACHI S4800) and atomic force microscopy (AFM, Veeco/DI). The XPS measurements were performed in ESCALAB 250Xi (Mg anode, 250 W, 14 kV), and the binding energy of the C 1*s* peak at 284.8 eV was taken as an internal pristine. XRD spectra of the prepared perovskite films were measured using powder X-ray diffraction (PXRD, Bruker Advance D8 X-ray diffractometer, Cu Kα radiation, 40 kV). Time-of-flight secondary-ion mass spectrometry (ToF-SIMS VI, IONTOF GmbH, Muenster, Germany) elemental depth profiling was used to probing the distribution of elements. UV–Vis spectra were collected using a Cary 500 UV–Vis–NIR spectrophotometer in air ambient environments. The steady-state PL and TRPL spectra of perovskite films were acquired by Fluorolog-3-p spectrophotometer and Endinburgh FLS890 spectrometer in air at room temperature, respectively. PL excitation wavelength was 380 nm. Solar cells were illuminated by a solar light simulator (Solar IV-150A, Zolix) and light intensity was calibrated by a standard Newport calibrated KG5-filtered Si reference cell. The *J*–*V* curves of devices were measured with Keithley 2400 digital sourcemeter under AM 1.5G irradiation (100 mW cm^−2^) at a scan rate of 0.15 V s^−1^ (voltages scan range: 0.3–1.5 V, voltage step of 10 mV) in air ambient environments. Devices were masked with a metal aperture to define the active area to be 0.0625 cm^2^. The steady-state photocurrent output of the best-performing devices was measured by biasing the device at MPP in air ambient environments. EIS were measured out using an electrochemical workstation (Parstat 2273, Princeton) in the frequency range of 1 MHz and 1 Hz under different positive bias voltages at dark conditions in air. The EQE was carried out on a Newport-74125 system (Newport Instruments) in air.

The CsPbI_2_Br films and unsealed devices were stored in air ambient environments with 15 ± 3% relatively humidity for long-term humidity test. White LED light illumination of 100 mW cm^**−**2^ for illumination stability tests of unencapsulated devices in nitrogen glovebox. The unencapsulated devices were baked on the hot plate at 85 °C in nitrogen glovebox for thermal stability test.

### Computational details

The first-principles DFT calculations were performed using the Vienna Ab initio Simulation Package code in this study^46–48^. The projector augmented wave method was used to describe the electron-ion interaction^49^, with valence electron structures of 5*s*^2^5*p*^6^6*s*^1^ for Cs (Cs_sv), 5*d*^10^6*s*^2^6*p*^2^ for Pb (Pb_d), 5*s*^2^5*p*^5^ for I, 4*s*^2^4*p*^5^ for Br, 3*s*^2^3*p*^4^ for S, 2*s*^2^2*p*^2^ for C, 2*s*^2^2*p*^3^ for N, and 1*s*^1^ for H. The exchange and correlation effects of the electron–electron were described by using the Perdew–Burke–Ernzerhof (PBE) functional of a generalized gradient approximation method^50^. The plane-wave kinetic energy cutoff was set at 520 eV to expand the smooth part of wave functions. All calculations were carried out on the thermodynamically stable (001) surface using a (1 × 2) five-layer slab model with the periodic boundary condition. A vacuum region of 20 Å was used to avoid the interaction between slabs. The Brillouin zone was sampled using Gamma-centered (5 × 3 × 1) *k*-point mesh for all surface calculations. Since traditional DFT calculations at the PBE level cannot correctly include the nonlocal van der Waals interactions, the calculations with dispersion corrections may affect the adsorption energies of small molecules^51,52^. In this regard, the DFT‐D3 method was adopted for dispersion corrections here^53^. During the geometry optimizations, the bottom two layers were fixed at the bulk position, whereas the top three atomic layers and the adsorbents were fully relaxed with the energy and force convergences <10^−5^ eV and 0.01 eV/Å, respectively.

The adsorption energy of each adsorbate [Δ*E*_ad_ (eV)] was calculated as follows:

2 $${\mathrm{{\Delta}}}E_{{\mathrm{ad}}} = E_{{\mathrm{ad}}/{\mathrm{surf}}} - E_{{\mathrm{surf}}} - E_{{\mathrm{ad}}}.$$

where *E*_ad_, *E*_surf_, and *E*_ad/surf_ are the energies of an adsorbate, the clean (001) surface and the surface with adsorbates, respectively. For the calculations of adsorbate molecules, a (20 × 20 × 20) Å^3^ unit cell and a *Γ*-only *k*-point grid were used.

## Supplementary information

Supplementary Information

## Data Availability

The data that support the plots within this paper are available in separate Supplementary Source Data Files in Supplementary information section. All other relevant data are available from the corresponding authors upon reasonable request.
