# Supplementary material for: Surface chelation of cesium halide perovskite by dithiocarbamate for efficient and stable solar cells
Source: Nat Commun. 2020 Aug 25;11:4237. doi: 10.1038/s41467-020-18015-5 (PMC7447778; doi:10.1038/s41467-020-18015-5)
Supplement: Supplementary file 1 — Supplementary Information [file 41467_2020_18015_MOESM1_ESM.pdf]

SUPPLEMENTARY INFORMATION

Surface chelation of cesium halide perovskite by dithiocarbamate for efficient and stable solar cells

Jingjing He[1], Junxian Liu[2], Yu Hou[1]*, Yun Wang[2]*, Shuang Yang[1]*, Hua Gui Yang[1]

[1]Key Laboratory for Ultrafine Materials of Ministry of Education, Shanghai Engineering Research Center of Hierarchical Nanomaterials, School of Materials Science and Engineering, East China University of Science and Technology, Shanghai 200237, China.

[2]Centre for Clean Environment and Energy, School of Environment and Science, Gold Coast Campus, Griffith University, Queensland 4222, Australia.

*Correspondence and requests for materials should be addressed to Shuang Yang (syang@ecust.edu.cn), Yun Wang (yun.wang@griffith.edu.au) and Yu Hou (yhou@ecust.edu.cn)

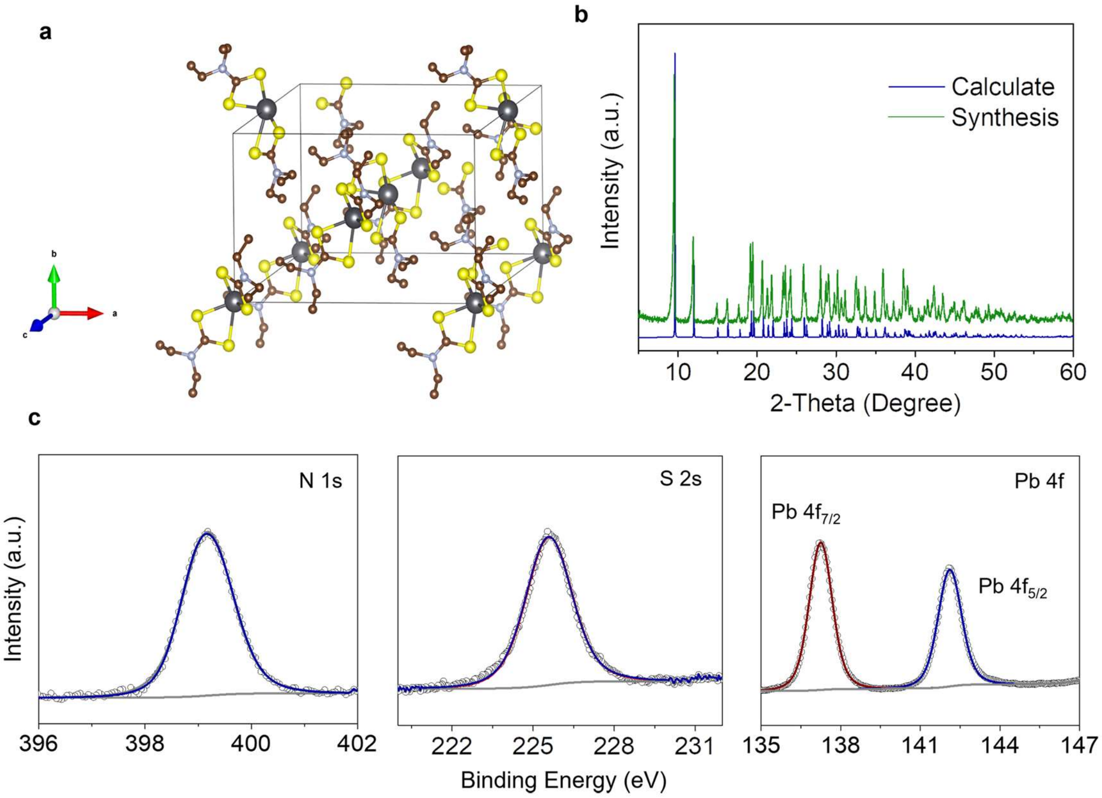

Supplementary Figure 1. Structure characterization of Pb(DDTC)$_2$. a, 3D structure of Pb(DDTC)$_2$ crystal. **b**, XRD patterns of the Pb(DDTC)$_2$ based on theoretical calculation (blue) and as-synthesis (green). **c**, XPS spectra of N 1s (left), S 2s (middle) and Pb 4f (right) of Pb(DDTC)$_2$.

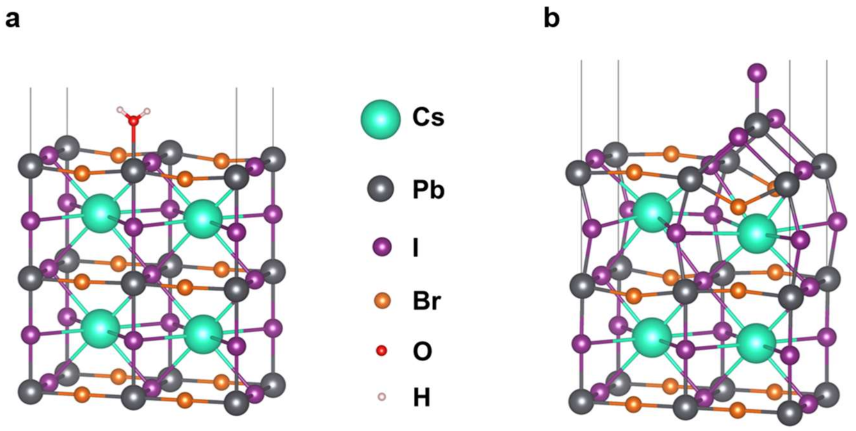

Supplementary Figure 2. Atomic structure of optimized $CsPbI_2Br$ (001) surface with adsorbed (**a**) H_2O and (**b**) PbI_2, respectively.

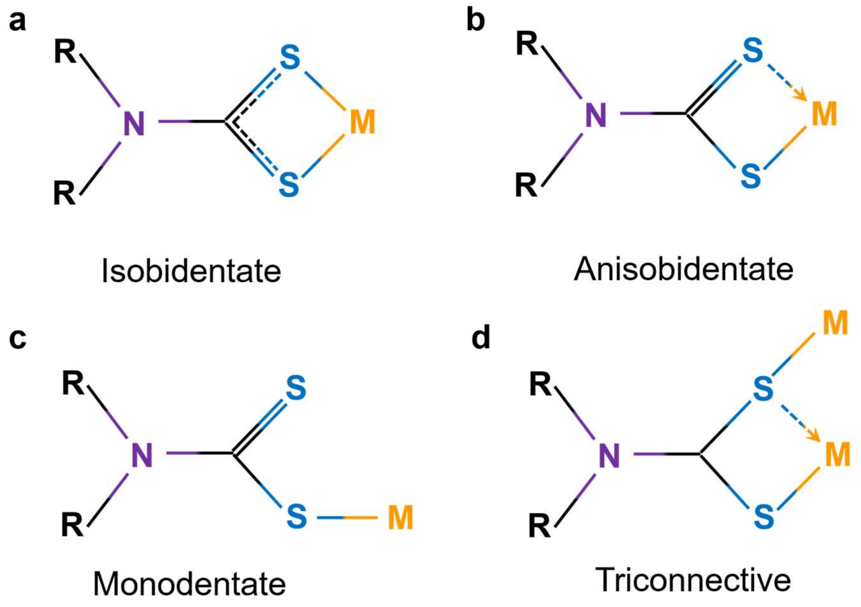

Supplementary Figure 3. Coordination modes of dithiocarbamate in the configurations of (**a**) isobidentate, (**b**) anisobidentate, (**c**) monodentate and (**d**) triconnective.

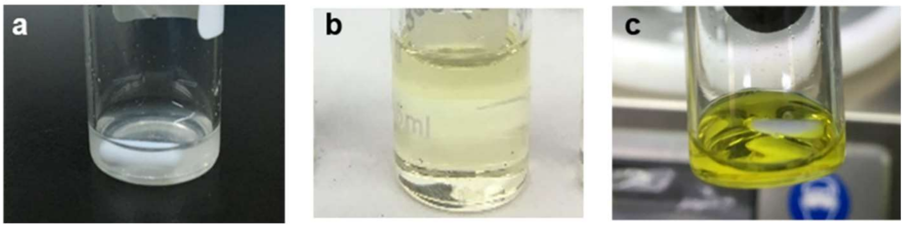

Supplementary Figure 4. Photographs of 0.02 M $Pb(DDTC)_2$ powers in (**a**) water, (**b**) DMSO and (**c**) $CsPbI_2Br$-DMSO solution at room temperature. $Pb(DDTC)_2$ powers are insoluble in water, but well dissolved in DMSO solution.

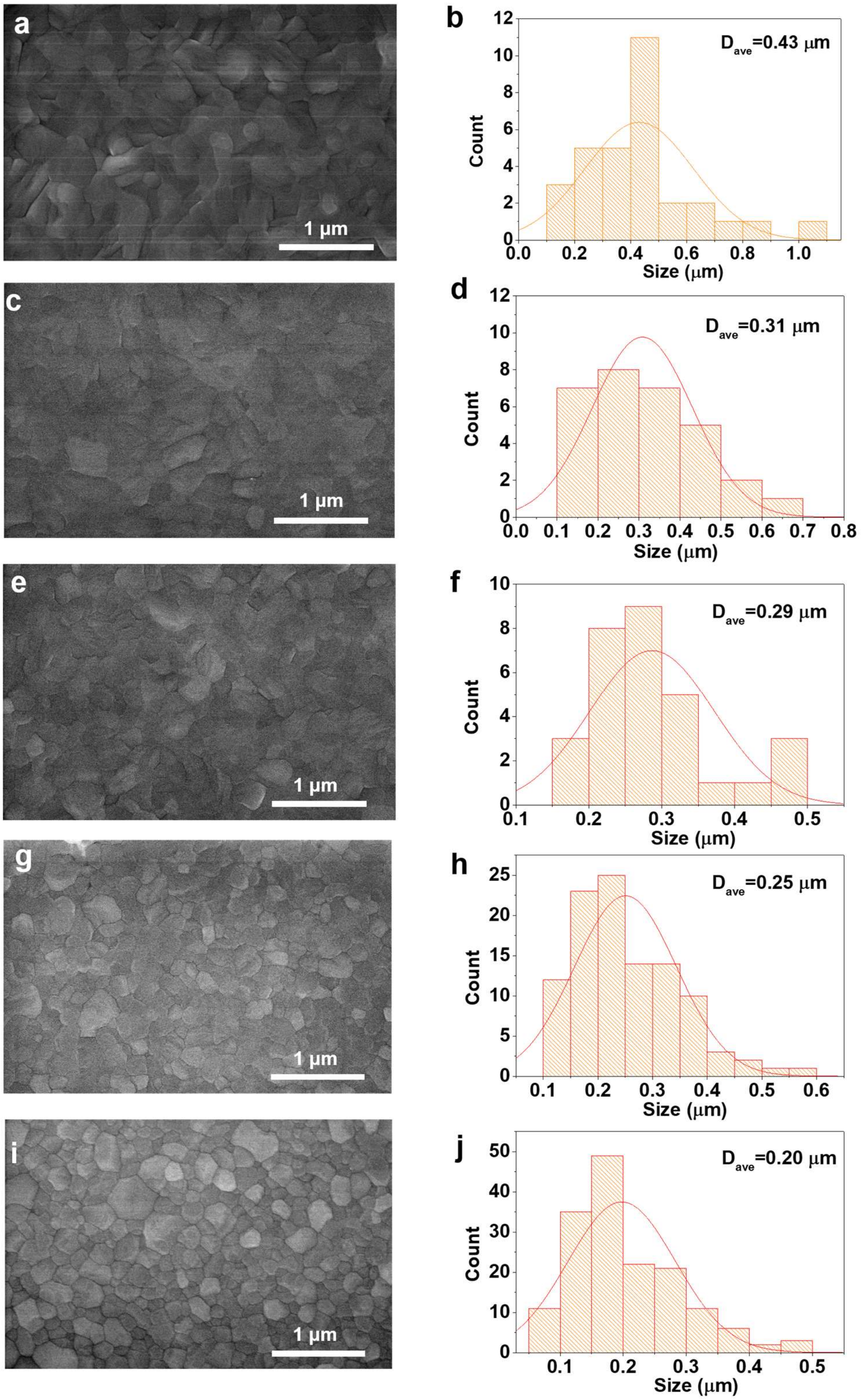

Supplementary Figure 5. Top view SEM images and corresponding statistical size distributions for the $CsPbI_2Br$ films with $Pb(DDTC)_2$ contents of (**a**,**b**) 0, (**c**,**d**) 0.005, (**e**,**f**) 0.010, (**g**,**h**) 0.015 and (**i**,**j**) 0.020 M, respectively. All films were deposited on glass/FTO/c-TiO_2 substrates.

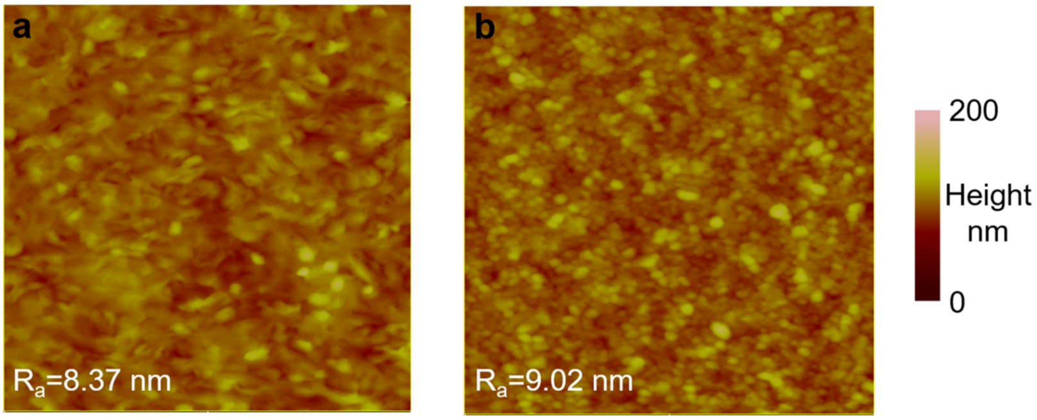

Supplementary Figure 6. AFM images of the (**a**) pristine and (**b**) chelated $CsPbI_2Br$ films. The concentration of $Pb(DDTC)_2$ used for the chelated sample is 0.015 M. Both perovskite films were deposited on the glass/FTO/c-TiO_2 substrates. The region of the AFM images is 5 × 5 μm.

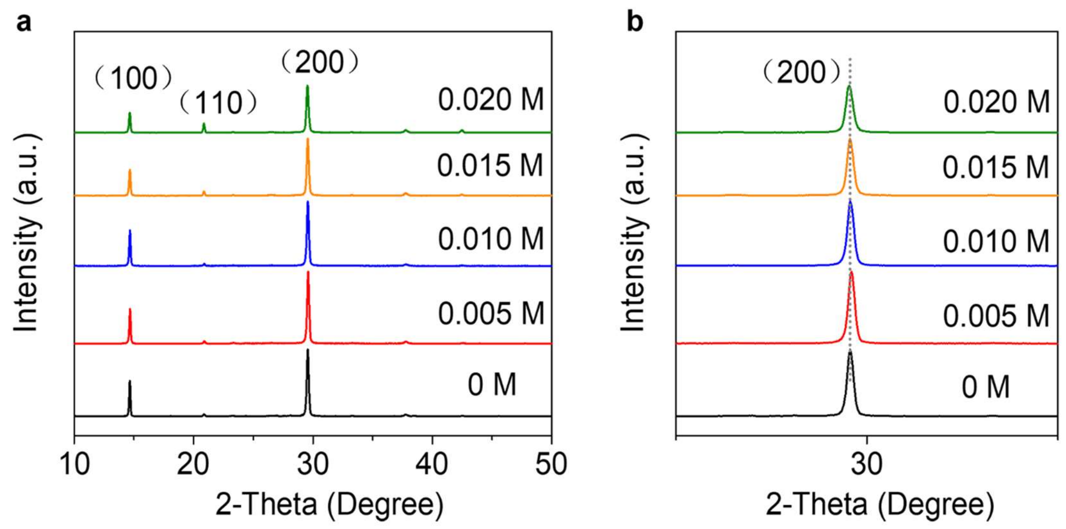

Supplementary Figure 7. Structural characterization of $CsPbI_2Br$ perovskite films. **a**, XRD patterns of the $CsPbI_2Br$ thin films with different concentration of $Pb(DDTC)_2$. **b**, Enlarged images of XRD patterns. All films were deposited on the glass/FTO/c-TiO_2 substrates.

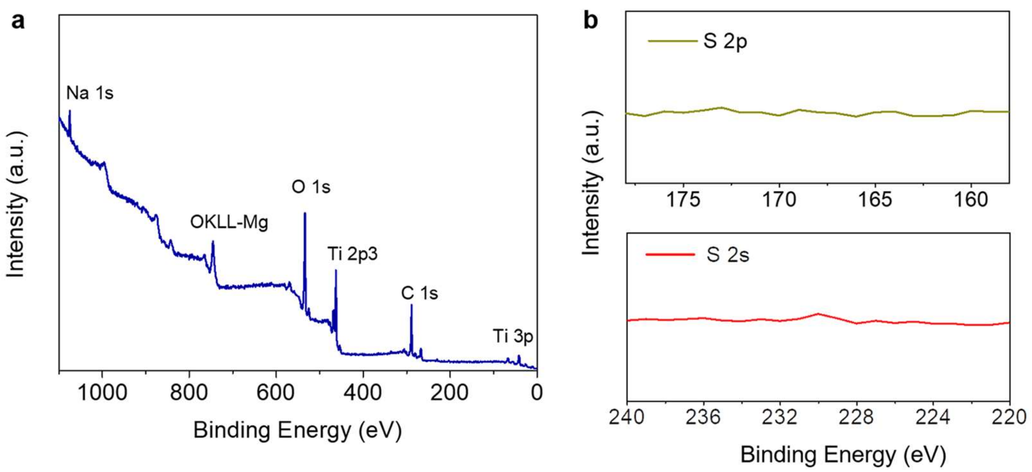

Supplementary Figure 8. XPS characterizations of the glass/FTO/c-TiO_2 substrate: (**a**) full spectrum and (**b**) S 2p and 2s spectra of glass/FTO/c-TiO_2 substrate.

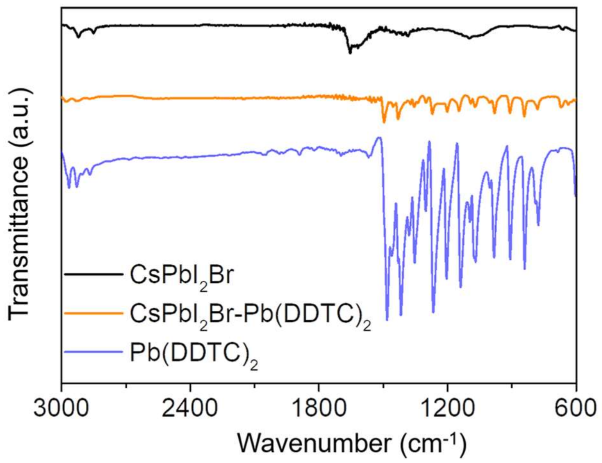

Supplementary Figure 9. Fourier transform infrared spectra of the pristine $CsPbI_2Br$ (black), chelated $CsPbI_2Br$ (orange), and $Pb(DDTC)_2$ (blue) powders. The concentration of $Pb(DDTC)_2$ used for the chelated sample is 0.02 M.

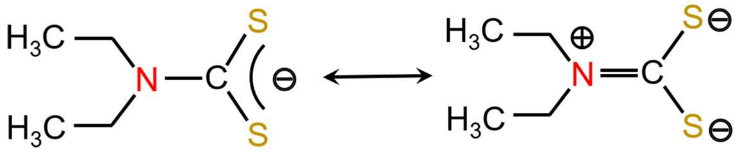

Supplementary Figure 10. Chemical structure of the dithiocarbamte (left) and thioureide (right).

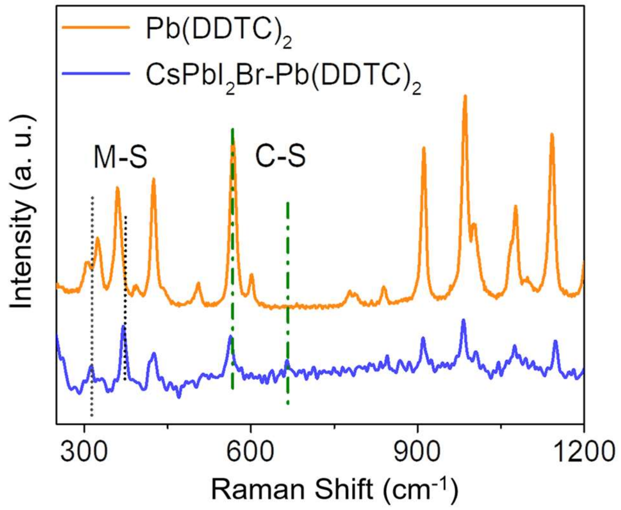

Supplementary Figure 11. Raman spectra of the $Pb(DDTC)_2$ (orange) and chelated $CsPbI_2Br$ (blue) powers. The concentration of $Pb(DDTC)_2$ used for the chelated sample is 0.02 M.

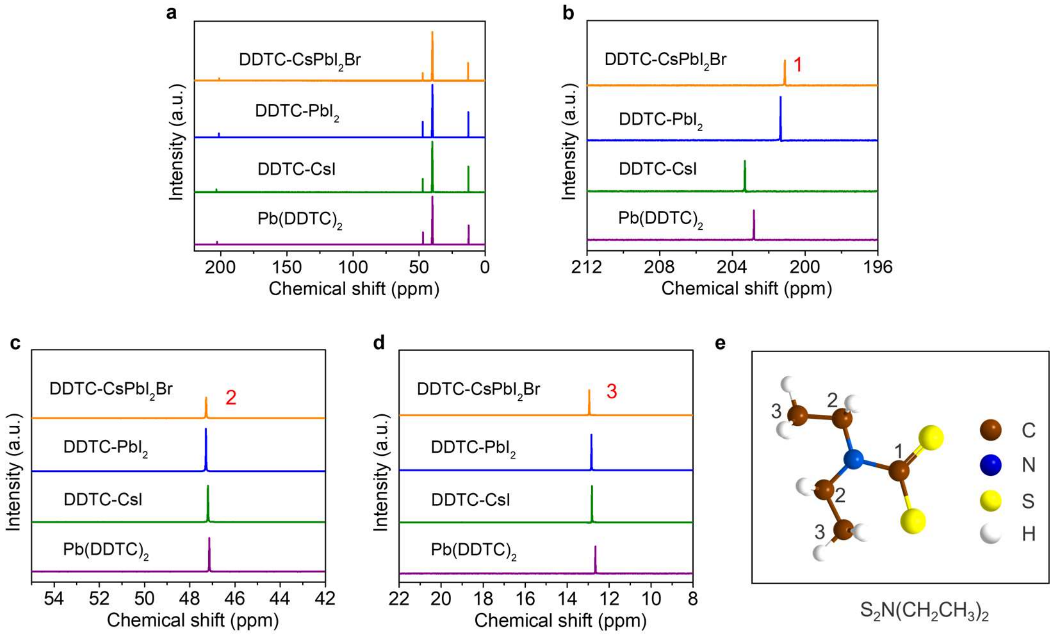

Supplementary Figure 12. Characterization of the interaction between $Pb(DDTC)_2$ and PbI_2 and $CsPbI_2Br$, respectively. **a**, Carbon-13 nuclear magnetic resonance (^{13}C NMR) spectra of $Pb(DDTC)_2$ (purple), $Pb(DDTC)_2$ with CsI (green), PbI_2 (blue) and $CsPbI_2Br$ (orange), respectively. **b**, **c**, **d**, Magnified ^{13}C NMR signals of carbon atom of –(NCS_2) group, methylene carbon atom and methyl carbon atom of the –$(CH_2CH_3)_2$ group, respectively. ^{13}C NMR signals of carbon atom of –(NCS_2) group are indexed with numbers in **e**. All samples were dissolved in deuterated DMSO-d_6 solution, with the concentration of $Pb(DDTC)_2$, PbI_2 and $CsPbI_2Br$ of 0.1, 1.0 and 1.0 M, respectively.

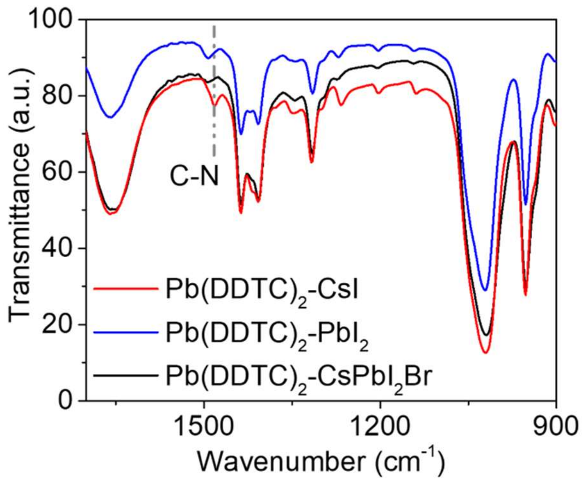

Supplementary Figure 13. Fourier transform infrared (FTIR) spectra of different precursors dissolution in DMSO solutions. The concertation of $Pb(DDTC)_2$, CsI, PbI_2 and $CsPbI_2Br$ is 0.02, 1.0, 1.0 and 1.0 M, respectively.

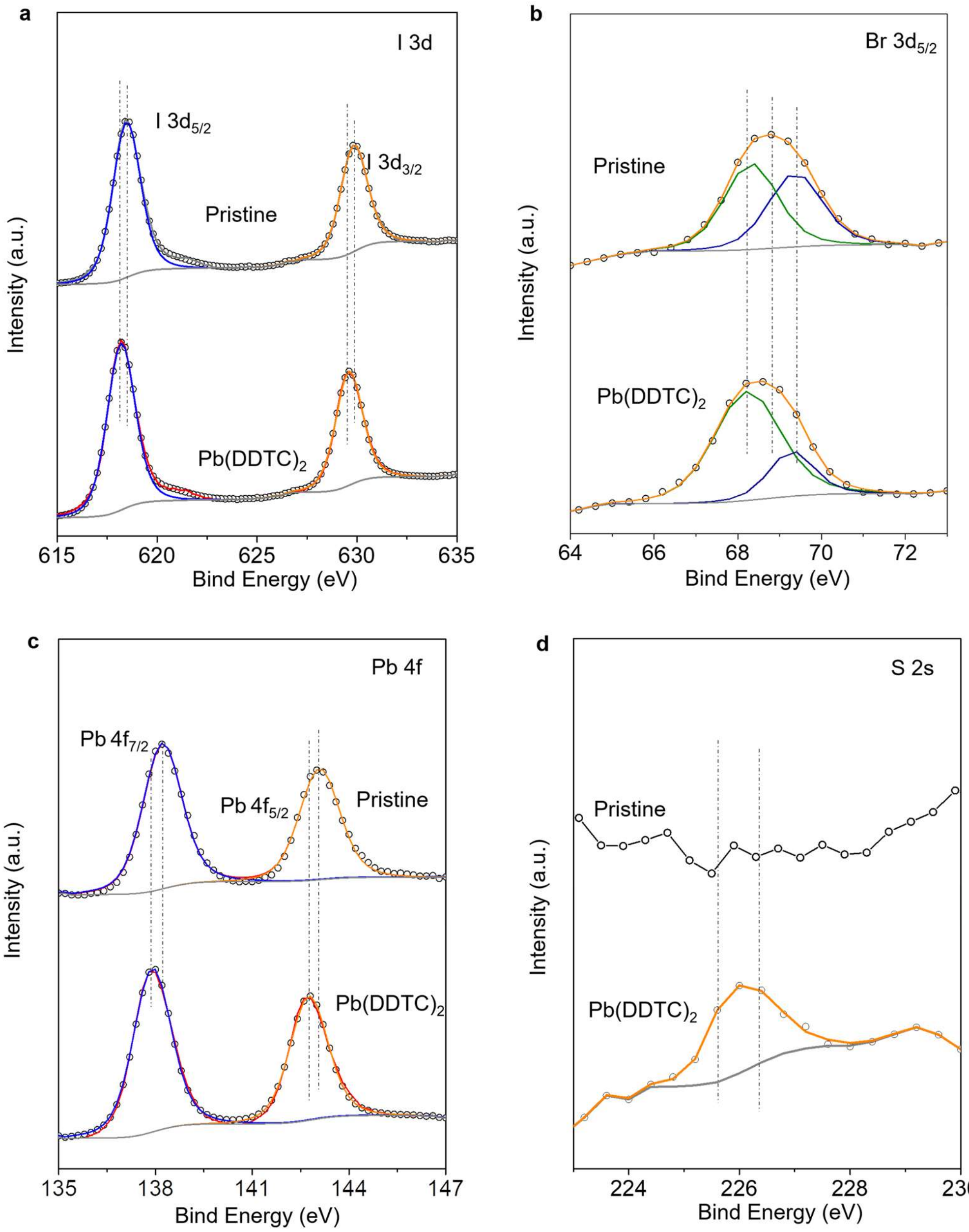

Supplementary Figure 14. XPS spectra of (**a**) I 3d, (**b**) Br $3d_{5/2}$, (**c**) Pb 4f and (**d**) S 2s of pristine and chelated ($Pb(DDTC)_2$) $CsPbI_2Br$ films. All films were deposited on glass/FTO/c-TiO_2 substrates.

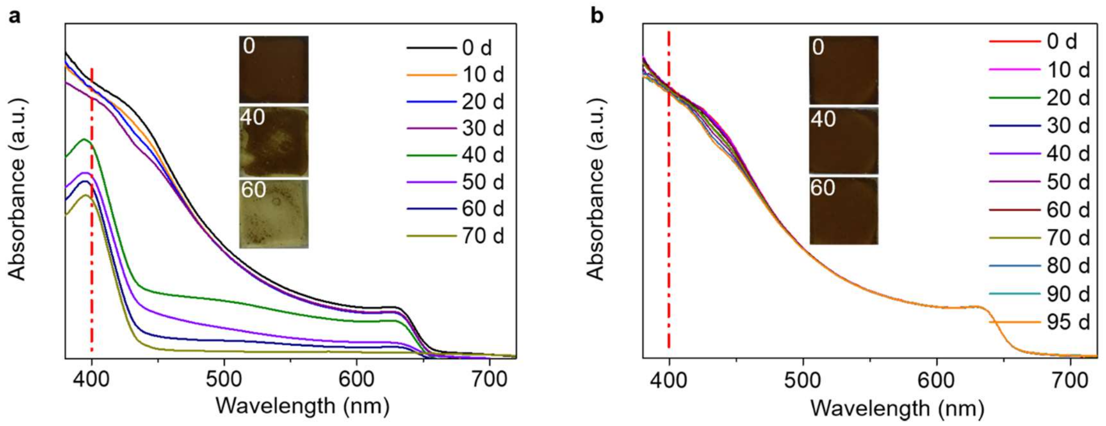

Supplementary Figure 15. Ultraviolet-visible (UV-Vis) absorption spectra of the (**a**) pristine and (**b**) chelated $CsPbI_2Br$ films. Insets are the photographs of corresponding perovskite films aged for 0, 40 and 60 days, respectively. All films were deposited on glass/FTO/c-TiO_2 substrates. The films were stored in ambient atmosphere with humidity of 15 ± 3% and measured at different time intervals. The chelated $CsPbI_2Br$ film contains the $Pb(DDTC)_2$ of 0.015 M.

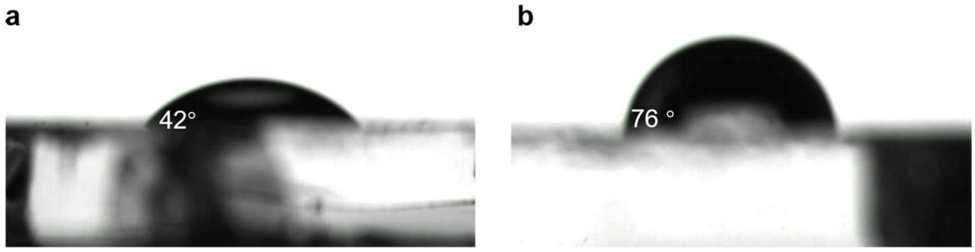

Supplementary Figure 16. Water contact angles of (**a**) pristine and (**b**) chelated $CsPbI_2Br$ films. The contact angles were captured at ~3 s after applying the drop of water. The concentration of $Pb(DDTC)_2$ used for the chelated sample is 0.015 M. All $CsPbI_2Br$ films were deposited on glass/FTO/c-TiO_2 substrates.

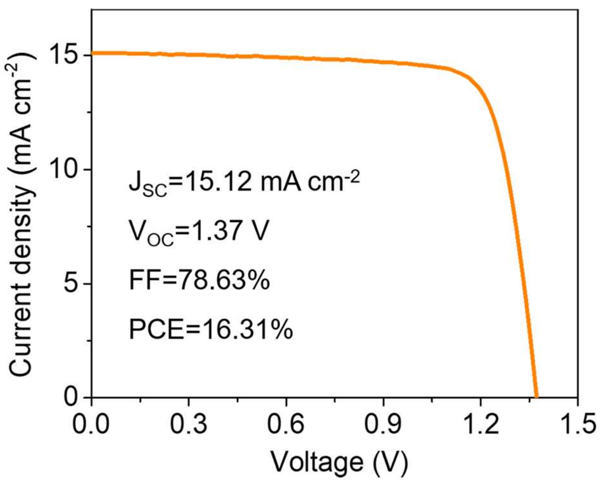

Supplementary Figure 17. J-V curve of device with the champion V_{OC} measured by reverse scan under standard AM 1.5G irradiation.

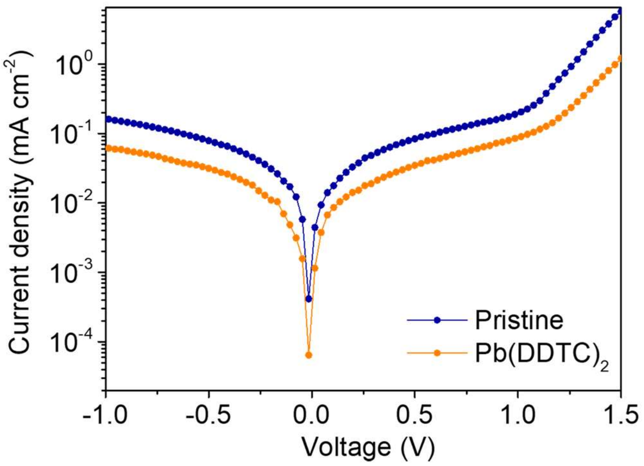

Supplementary Figure 18. Dark J-V curves of the pristine (blue) and chelated (orange) $CsPbI_2Br$ solar cells.

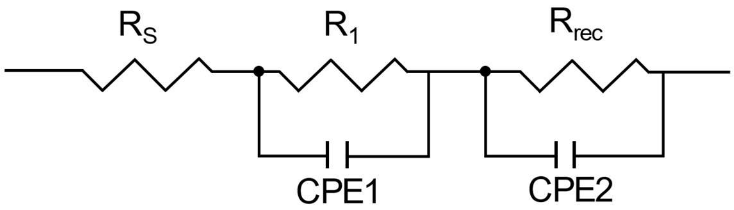

Device	R_s (Ω)	R_1 (Ω)	R_{rec} (Ω)	CPE1-T(F)	CPE2-T(F)
Pristine	25.9	87.5	1737.5	1.124E-8	4.317E-9
$Pb(DDTC)_2$	29.9	72.7	7495.3	1.043E-8	8.725E-9

Supplementary Figure 19. Equivalent circuit model and fitting parameters for the EIS spectra at bias voltage of 1.0 V. R_s, R_1, R_{rec} and CPE are series resistance, transport resistance, recombination resistance and the chemical capacitance, respectively.

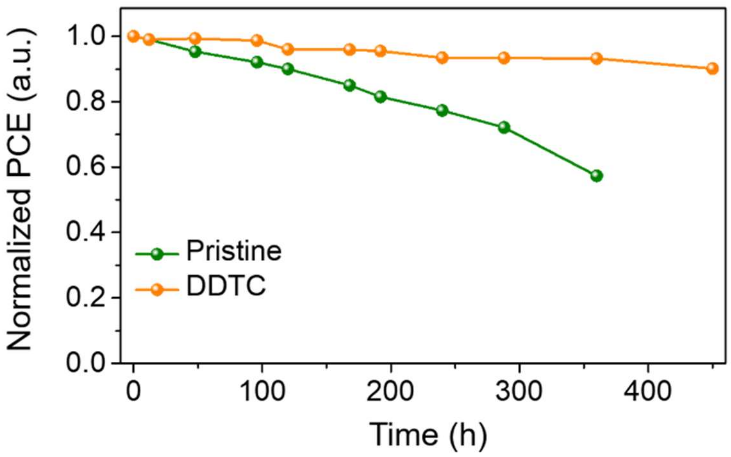

Supplementary Figure 20. Thermal stability test. Normalized PCEs of the pristine (green) and chelated (orange) $CsPbI_2Br$ device heated at 85 °C in N_2 atmosphere without encapsulation. The device structure is FTO/c-TiO_2/perovskite/P3HT/Au.

Supplementary Table 1. Photovoltaic parameters for typical $CsPbI_2Br$ devices based on different concentration of $Pb(DDTC)_2$. All J-V curves were measured under simulated AM 1.5G illumination with a reverse scan rate of 0.15 V s^{-1}.

Concentration (M)	J_{SC} (mA cm^{-2})	V_{OC} (V)	FF (%)	PCE (%)
0	15.88	1.19	77.28	14.54
0.005	15.79	1.28	78.26	15.82
0.010	15.72	1.31	79.19	16.34
0.015	15.68	1.34	78.77	16.57
0.020	15.63	1.30	75.52	15.31

Supplementary Table 2. Photovoltaic parameters for the champion $CsPbI_2Br$ devices with reverse and forward scanning directions. All J-V curves were measured under simulated AM 1.5G illumination with a reverse scan rate of 0.15 V s^{-1}.

–	J_{SC} (mA cm^{-2})	V_{OC} (V)	FF (%)	PCE (%)
Pristine-reverse	15.43	1.25	77.08	14.80
Pristine-forward	15.30	1.20	74.47	13.70
Chelated-reverse	15.78	1.34	80.52	17.03
Chelated- forward	15.75	1.32	79.83	16.60

Supplementary Table 3. Comparison of the reported photovoltaics performance of $CsPbI_2Br$ perovskite solar cells.

Device Structure	J_{SC} (mA cm^{-2})	V_{OC} (V)	FF (%)	PCE (%)	Ref.
FTO/TiO_2/$CsPbI_2Br$/P3HT/Ag	15.78	1.34<br>1.37 (Highest)	80.52	17.03	This work
ITO/SnO_2/BPQDs@$CsPbI_2Br$/Spiro-OMeTAD/Au	15.86	1.25	78.00	15.47	1
ITO/TiO_2-Cl/$CsPbI_2Br$/PDCBT/MoO_x/Au	15.53	1.24	85.2	16.41	2
FTO/c-TiO_2/m-TiO_2/$CsPb_{1-x}Ba_xI_2Br$/Spiro-OMeTAD/Au	14.00	1.28<br>1.33	78.20	14.00	3
FTO/NiOx/$CsPbI_2Br$/ZnO@C_{60}/Ag	15.87	1.23	78.00	15.19	4
ITO/SnO_2/$CsPbI_2Br$/Spiro-OMeTAD/Au	16.31	1.18	78.80	15.17	5
FTO/ZnO@SnO_2/$CsPbI_2Br$/Sprio-OMeTAD/MoO_3/Ag	16.45	1.11	79.00	14.35	6
FTO/c-TiO_2/$CsPbI_2Br$/Spiro-OMeTAD/Au	16.82	1.15	75.73	14.69	7
ITO/SnO_2/$CsPbI_2Br$/PTAA/Au	16.85	1.23	80.00	16.58	8
ITO/c-TiO_2/$CsPbI_2Br$/Spiro-OMeTAD/Au	16.79	1.23	77.81	16.07	9
FTO/c-TiO_2/m-TiO_2/Eu-$CsPbI_2Br$/Spiro-OMeTAD/Au	14.63	1.22	76.60	13.71	10
ITO/SnO_2/PN4N/$CsPbI_2Br$/PDCBT/MoO_3 /Ag	15.30	1.30	81.50	16.20	11

Supplementary References

1. Gong, X. et al. Black phosphorus quantum dots in inorganic perovskite thin films for efficient photovoltaic application. *Sci. Adv.* **6**, eaay5661 (2020).

2. Xue, D.-J. et al. Regulating strain in perovskite thin films through charge-transport layers. *Nat. Commun.* **11**, 1514 (2020).

3. Xiang, W. et al. Ba-induced phase segregation and band gap reduction in mixed-halide inorganic perovskite solar cells. *Nat. Commun.* **10**, 4686 (2019).

4. Liu, C. et al. Tailoring C_{60} for efficient inorganic $CsPbI_2Br$ perovskite solar cells and modules. *Adv. Mater.* **32**, 1907361 (2020).

5. Liu, S.-C. et al. Investigation of oxygen passivation for high-performance all-inorganic perovskite solar cells. *J. Am. Chem. Soc.* **141**, 18075−18082 (2019).

6. Li, Z. et al. Core-Shell ZnO@SnO_2 Nanoparticles for efficient inorganic perovskite solar cells. *J. Am. Chem. Soc.* **141**, 17610−17616 (2019).

7. Fan, Y. et al. Scalable ambient fabrication of high-performance $CsPbI_2Br$ solar Cells. *Joule* **3**, 2485−2502, (2019).

8. Xue, J. et al. Crystalline liquid-like behavior: surface-induced secondary grain growth of photovoltaic perovskite thin film. *J. Am. Chem. Soc.* **141**, 13948−13953 (2019).

9. Chen, W. et al. Precise control of crystal growth for highly efficient $CsPbI_2Br$ perovskite solar cells, *Joule* **3**, 191−204 (2019).

10. Xiang, W. et al. Europium-doped $CsPbI_2Br$ for stable and highly efficient inorganic perovskite solar cells. *Joule* **3**, 205−214 (2019).

11. Tian, J. et al. Dual interfacial design for efficient $CsPbI_2Br$ perovskite solar cells with improved photostability. *Adv. Mater.* **31**, 1901152 (2019).
